# Incidence of and risk factors for community-associated *Clostridium difficile *infection: A nested case-control study

**DOI:** 10.1186/1471-2334-11-194

**Published:** 2011-07-15

**Authors:** Jennifer L Kuntz, Elizabeth A Chrischilles, Jane F Pendergast, Loreen A Herwaldt, Philip M Polgreen

**Affiliations:** 1 Center for Health Research, Kaiser Permanente Northwest, Portland, OR, USA; 2 College of Public Health, University of Iowa, Iowa City, IA, USA; 3 Carver College of Medicine, University of Iowa, Iowa City, IA, USA

## Abstract

**Background:**

*Clostridium difficile *is the most common cause of nosocomial infectious diarrhea in the United States. However, recent reports have documented that *C. difficile *infections (CDIs) are occurring among patients without traditional risk factors. The purpose of this study was to examine the epidemiology of CA-CDI, by estimating the incidence of CA-CDI and HA-CDI, identifying patient-related risk factors for CA-CDI, and describing adverse health outcomes of CA-CDI.

**Methods:**

We conducted a population-based, retrospective, nested, case-control study within the University of Iowa Wellmark Data Repository from January 2004 to December 2007. We identified persons with CDI, determined whether infection was community-associated (CA) or hospital-acquired (HA), and calculated incidence rates. We collected demographic, clinical, and pharmacologic information for CA-CDI cases and controls (i.e., persons without CDI). We used conditional logistic regression to estimate the odds ratios (ORs) for potential risk factors for CA-CDI.

**Results:**

The incidence rates for CA-CDI and HA-CDI were 11.16 and 12.1 cases per 100,000 person-years, respectively. CA-CDI cases were more likely than controls to receive antimicrobials (adjusted OR, 6.09 [95% CI 4.59-8.08]) and gastric acid suppressants (adjusted OR, 2.30 [95% CI 1.56-3.39]) in the 180 days before diagnosis. Controlling for other covariates, increased risk for CA-CDI was associated with use of beta-lactam/beta-lactamase inhibitors, cephalosporins, clindamycin, fluoroquinolones, macrolides, and penicillins. However, 27% of CA-CDI cases did not receive antimicrobials in the 180 days before their diagnoses, and 17% did not have any traditional risk factors for CDI.

**Conclusions:**

Our study documented that the epidemiology of CDI is changing, with CA-CDI occurring in populations not traditionally considered "high-risk" for the disease. Clinicians should consider this diagnosis and obtain appropriate diagnostic testing for outpatients with persistent or severe diarrhea who have even remote antimicrobial exposure.

## Background

*Clostridium difficile *is the most common cause of nosocomial infectious diarrhea in the United States. Several reports indicate that the incidence and the severity of *C. difficile *infections (CDI) are increasing [1-3], possibly related to the new virulent BI/NAP1 strain [4]. Investigators have identified numerous risk factors for hospital-acquired CDI (HA-CDI) (e.g., antimicrobial use, older age, underlying diseases) [5-9]. However, recent published reports have described CDI cases in people without traditional risk factors [10-12], including people without recent exposures to antimicrobials. These reports suggest that community-associated CDI (CA-CDI) cases are occurring in persons who are younger, have fewer comorbidities, and less exposure to healthcare than persons with HA-CDI [10-15].

Few large studies have been conducted to identify risk factors for CDI in the community-setting, and investigators have not determined if or to what extent the epidemiology of CA-CDI differs from that of HA-CDI. Furthermore, most studies of CA-CDI in the United States are based on brief periods of voluntary surveillance in limited geographic areas and in targeted populations [12,15,16]. The purpose of this study was to examine the epidemiology of CA-CDI in a broad population. Specifically, this study estimates the incidence of CA-CDI and HA-CDI within an employer-based, insured population covering two states, identifies patient-related risk factors for CA-CDI, and describes adverse health outcomes of CA-CDI.

## Methods

### Design Overview

We conducted a retrospective, nested, case-control study using the Wellmark Data Repository (Data Repository), which is housed at the University of Iowa College of Public Health, to identify persons with CDI from January 1, 2004 to December 31, 2007. The Data Repository is a limited, longitudinal data set consisting of de-identified healthcare claims for members and their covered family members who are fully-insured through policies underwritten by Wellmark, the largest provider of health insurance in Iowa and South Dakota. This study was approved by the University of Iowa Institutional Review Board.

We examined insurance claims for inpatient, outpatient, home health, extended care/skilled nursing, and outpatient pharmacy healthcare services provided to members with health and prescription drug coverage. These data included insurance coverage, demographic information, diagnosis codes, procedure codes, dates of service and, outpatient pharmacy data including fill dates and drug-days supplied.

### Identification of Case and Control Patients

We identified cases as persons with a primary or secondary diagnosis of ICD-9 code 008.45 for 'Infection due to *Clostridium difficile*' listed on an inpatient or outpatient insurance claim. Case subjects were required to have a minimum of 12 months of continuous health and pharmacy insurance coverage before their diagnosis and not have a history of healthcare claims from a long-term care facility during the 6 months before their diagnoses. Only the first *C. difficile *diagnosis was included. The diagnosis date was defined as the date on which the ICD-9 code for CDI first appeared on a claim.

A case of CA-CDI either had: (1) a diagnosis of CDI in the outpatient setting with no history of hospital discharge in the 12 weeks before diagnosis, or (2) a primary diagnosis upon hospital admission and no history of hospital discharge in the 12 weeks before diagnosis. A case of HA-CDI had either: (1) a secondary diagnosis during hospitalization; (2) a primary diagnosis upon admission to a hospital with a history of hospital discharge in the 4 weeks before diagnosis; or, (3) a diagnosis of CDI in the outpatient setting with a history of hospital discharge in the 4 weeks before diagnosis. All other cases were defined as indeterminate (i.e., did not meet the definitions of CA-CDI or HA-CDI).

For each CA-CDI case subject, ten control subjects were randomly selected. Members were eligible to serve as controls if they met inclusion criteria for cases (i.e., minimum of 12 months of continuous health and pharmacy insurance coverage and no history of healthcare claims from a long-term care facility in the prior 6 months), but had not been diagnosed with CDI on or before the date of diagnosis for a corresponding case [17]. A member could be a control subject and subsequently a case subject if CDI was diagnosed later. The index date for each control was the diagnosis date for the matched case. No further matching criteria were imposed.

### Assessment of Exposures to Antimicrobial Agents and Gastric Acid Suppressants among CA-CDI Cases and Controls

We examined outpatient prescription use of antimicrobials among CA-CDI cases and controls in the 180 days before the diagnosis or index date. Prescription medications were identified through National Drug Codes (NDCs) on outpatient prescription drug claims. We examined exposure to specific antimicrobial classes or agents (aminoglycosides, beta-lactam/beta-lactamase inhibitors, cephalosporins, clindamycin, fluoroquinolones, macrolides, penicillins, sulfonamides, tetracyclines, and intravenous vancomycin), the total number of different antimicrobial agents received, and the timing of antimicrobial use in relation to the diagnosis or index dates. We categorized the timing of the subjects' most recent antimicrobial exposures as use during the following mutually exclusive intervals before the diagnosis or index dates (calculated from the last prescription's fill date and the days of antimicrobial supplied): 1 to 30 days, 31 to 60 days, 61 to 90 days, 91 to 120 days, 121 to 150 days, and 151 to 180 days. We did not examine use of topical or ophthalmic antimicrobials. In addition, we did not include use of metronidazole or oral vancomycin as possible risk factors, because they are used to treat CDI.

We also examined use of prescription gastric acid suppressants--proton pump inhibitors and histamine-2 receptor antagonists. Proton pump inhibitors included lansoprazole, omeprazole, pantoprazole, and rabeprazole. Histamine-2 receptor antagonists included cimetidine, and ranitidine. We categorized use of these medications as "never-used" or "ever-used" in the 180 days before the diagnosis or index dates.

### Assessment of Comorbidity and Healthcare Utilization among CA-CDI Cases and Controls

We used the Charlson Comorbidity Index to measure underlying comorbidity among CA-CDI cases and their controls [18,19]. Charlson comorbid conditions were considered "present" if the corresponding ICD-9 code was listed as a primary or secondary diagnosis on one inpatient claim or on two outpatient claims occurring 30 or more days apart in the year before the diagnosis or index dates [20]. We identified hospitalizations in the year before the diagnosis or index dates. We also determined whether or not patients had inflammatory bowel disease (i.e., Crohn's disease [ICD-9 codes: 555.0-555.9) and ulcerative colitis [ICD-9 codes: 556-556.9]).

### Identification of Adverse Outcomes among CA-CDI Cases

Potential adverse outcomes following CA-CDI included colectomies, subsequent hospitalizations for CDI, and relapse of infection. We determined whether persons with CA-CDI had colectomies (ICD-9 procedure codes: partial/subtotal [45.79], cecal [45.72], left colon [45.75], multiple segmental [45.71], right colon [45.73], sigmoid [45.76], subtotal [45.8], and transverse colon [45.74]) during the 180 days following diagnosis [21]. We defined hospitalization related to CDI as an admission with a primary diagnosis of CDI occurring on the date of diagnosis of CA-CDI or during the following 8 weeks [22]. We considered claims for metronidazole or oral vancomycin prescriptions submitted after claims for the initial therapy and within 180 days of the diagnosis date to be markers for CDI recurrence or relapse.

### Statistical Analyses

The incidence rates for CA-CDI and HA-CDI were the number of incident cases per 100,000 person-years of observation time. The denominator data were calculated based on duration of insurance coverage for each person in the Data Repository population for each year.

We calculated summary statistics for demographic characteristics, healthcare utilization, comorbid conditions, and medication among CA-CDI cases and controls. We used conditional logistic regression to estimate odds ratios (ORs) for the associations between CA-CDI and any antimicrobial use, use of individual antimicrobials, timing of antimicrobial use, number of antimicrobial agents prescribed, and use of gastric acid suppressants, while adjusting for covariates including comorbidity, inflammatory bowel disease (IBD), hospitalizations, age (in categories), and gender. In separate models, we estimated the ORs for the associations between CA-CDI and use of each antimicrobial class and also CA-CDI and the number of different antimicrobials. Because aminoglycosides and intravenous vancomycin were used infrequently, we excluded them from models for individual agents. When we assessed timing of antimicrobial use, we considered 'no antimicrobial use in the prior 180 days' to be the reference group. We used SAS version 9.2 (SAS Institute Inc., Cary, NC) for all analyses.

## Results

During the study period, we identified 684 cases of CDI, of which, 304 were CA-CDI, 338 were HA-CDI, and 42 were indeterminate CDI. The overall incidence rates for CA-CDI and HA-CDI were 11.16 and 12.41 cases per 100,000 person-years, respectively. Year-specific incidence rates are shown in Table 1.

**Table 1:** Number of cases and incidence rates of community-associated and hospital-acquired *C. difficile *infection, 2004-2007

Year	Total Person-years	Number of CA-CDI Cases	CA-CDI Incidence Rate*	Number of HA-CDI Cases	HA-CDI Incidence Rate*
2004	667,113	62	9.29	85	12.71
2005	673,630	84	12.47	76	11.28
2006	666,127	74	11.11	84	12.61
2007	716,265	84	11.76	93	12.98

^* ^Incidence rates expressed as the number of cases per 100,000 person-years.

The case-control study included 304 CA-CDI cases and 3040 controls. Baseline characteristics of case and control patients are shown in Table 2. The majority of the study subjects were between the ages of 19 and 64 years (76% of cases and 69% of controls), but CA-CDI cases were significantly older than control subjects. CA-CDI cases (11%) were more likely than controls (3%) to be hospitalized in the previous year. Cases had more Charlson comorbidities, although only 11% of cases and 4% of controls had Charlson indices of 1 or greater.

**Table 2:** Analysis of risk factors for community-associated *C. difficile *infection

Variable	CA-CDI Cases (N = 304)	Controls (N = 3040)	Unadjusted OR (95% CI)	Adjusted OR (95% CI)*
Age in Years (by category)				
<18 years	45 (14.80)	814 (26.78)	reference	reference
19 to 49 years	125 (41.12)	1296 (42.63)	0.94 (0.74, 1.19)	1.92 (1.32, 2.78)
50 to 64 years	106 (34.87)	803 (26.41)	1.49 (1.16, 1.91)	2.36 (1.59, 3.49)
65 to 74 years	18 (5.92)	92 (3.03)	2.03 (1.21, 3.43)	3.38 (1.73, 6.57)
≥75 years	10 (3.29)	35 (1.15)	2.90 (1.43, 5.90)	2.49 (1.01, 6.12)
Gender (female)	184 (60.53)	1570 (51.64)	1.44 (1.1, 1.83)	1.24 (0.95, 1.61)
History of Hospitalization in Previous Year	33 (10.86)	103 (3.39)	3.47 (2.30, 5.23)	1.60 (0.99, 2.60)
Charlson Comorbidity Index [Mean (SD)]	0.17 (0.62)	0.05 (0.27)	2.03 (1.55, 2.64)	1.33 (0.98, 1.79)
Inflammatory Bowel Disease	12 (3.95)	4 (0.13)	30.0 (9.68, 93.02)	41.89 (11.83, 148.35)
Antimicrobial Use				
None	82 (26.97)	2120 (69.74)	reference	reference
Any	222 (73.03)	920 (30.26)	6.12 (4.70, 7.98)	6.09 (4.59, 8.08)
Gastric Acid Suppressant Use^†^				
None	249 (81.91)	2883 (94.84)	reference	reference
Any	55 (18.09)	157 (5.16)	4.07 (2.91, 5.69)	2.30 (1.56, 3.39)

NOTE. Data are number (%) of patients, unless otherwise stated.

^* ^Adjusted for all other covariates

^† ^Includes proton pump inhibitors and histamine-2 receptor antagonists.

CA-CDI cases were more likely than controls to receive antimicrobials in the previous 180 days (adjusted OR 6.09, 95% CI 4.59-8.08) (Table 2). After adjusting for all covariates including antimicrobial use; age 19 years and older (with ≤ 18 years as the reference group), IBD, and gastric acid suppressant use significantly increased the risk of CA-CDI. Eighteen percent of CA-CDI cases and 5% of controls received gastric acid suppressants in the 180 days before their diagnosis or index dates (adjusted OR 2.30, 95% CI 1.56-3.39) (Table 2), and 84% of persons with CA-CDI who received gastric acid suppressants also received one or more antimicrobial agents (data not shown).

After controlling for other covariates, increased risk for CA-CDI was associated with use of beta-lactam/beta-lactamase inhibitors, cephalosporins, clindamycin, fluoroquinolones, macrolides, and penicillins (Table 3). The risk for CA-CDI was highest for antimicrobial use during the 30-days before diagnosis and remained significantly elevated for antimicrobial use as early as 150 days before diagnosis (Table 3). Finally, each antimicrobial agent prescribed significantly increased the risk for CA-CDI (Table 3).

**Table 3:** Association between antimicrobial use in the previous 180 days and community-associated *C. difficile *infection

	CA-CDI Cases	Controls	Unadjusted OR	Adjusted OR
	(N = 304)	(N = 3040)	(95% CI)	(95% CI)*
Antimicrobial Drug/Class^†^				
Beta-lactam/beta-lactamase inhibitors	46 (15.13)	95 (3.12)	5.58 (3.79, 8.20)	5.10 (3.26, 8.00)
Cephalosporins	75 (24.67)	230 (7.57)	4.06 (3.02, 5.47)	3.11 (2.17, 4.45)
Clindamycin	35 (11.51)	26 (0.86)	15.65 (9.09, 26.95)	13.00 (7.03, 24.04)
Fluoroquinolones	67 (22.04)	94 (3.09)	8.33 (5.94, 11.67)	4.91 (3.28, 7.35)
Macrolides	61 (20.07)	300 (9.87)	2.27 (1.68, 3.07)	2.19 (1.54, 3.11)
Penicillins	50 (16.45)	291 (9.57)	1.86 (1.34, 2.58)	1.72 (1.17, 2.54)
Sulfonamides	16 (5.26)	52 (1.71)	3.16 (1.79, 5.60)	1.58 (0.79, 3.15)
Tetracyclines	11 (3.62)	78 (2.57)	1.43 (0.75, 2.71)	0.94 (0.43, 2.04)

Timing of Antimicrobial Use				
No Use	82 (26.97)	2120 (69.74)	reference	reference
Within 1-30 Days	141 (46.38)	304 (10.00)	12.06 (8.88, 16.36)	13.02 (9.37, 18.09)
Within 31-60 Days	36 (11.84)	148 (4.87)	6.25 (4.06, 9.63)	5.84 (3.68, 9.28)
Within 61-90 Days	15 (4.93)	151 (4.97)	2.50 (1.40, 4.47)	2.30 (1.24, 4.25)
Within 91-120 Days	17 (5.59)	144 (4.74)	2.84 (1.63, 4.93)	2.30 (1.27, 4.17)
Within 121-150 Days	9 (2.96)	97 (3.19)	2.30 (1.12, 4.73)	2.77 (1.29, 5.95)
Within 151-180 Days	4 (1.32)	76 (2.50)	1.39 (0.50, 3.91)	1.17 (0.40, 3.41)

Number of Antimicrobials [Mean (SD)]	1.26 (1.10)	0.39 (0.68)	2.72 (2.40, 3.09)	2.74 (2.38, 3.15)

NOTE. Data are presented as the number (%) of patients, unless otherwise stated.

^* ^Adjusted for age, gender, history of hospitalization, Charlson Comorbidity Index, inflammatory bowel disease, and gastric acid suppressant use

^† ^Antimicrobial classes were entered in a multivariable model as a series of indicator variables (i.e., ORs are adjusted for the use of other classes).

We performed a sensitivity analysis to examine the relationship between risk factors and onset of CA-CDI after we redefined the diagnosis date as the date of the first: (1) CDI diagnosis code, (2) prescription for oral vancomycin or antimotility medication, or (3) diagnosis code for non-specific diarrheal disease. When we utilized this approach, 122 cases retained their original diagnosis dates based on ICD-9 codes, while 177 cases had revised diagnosis dates based on prescription medication use or diagnosis of diarrheal disease. The results of this analysis, which included cases with original and revised diagnosis dates and their controls, were essentially the same as those reported in Table 3.

No one with CA-CDI required a colectomy. Seventy-seven CA-CDI cases were admitted (79 admissions) with the primary diagnosis of ICD-9 code 008.45 entered on the diagnosis date or within 8 weeks, for a hospitalization rate of 25.3%. Of the 77 first-time admissions, sixty-three (81.8%) occurred on the date of diagnosis, 10 (12.7%) occurred 1 day after the CDI diagnosis, and 1 each occurred 4, 20, 24, and 30 days after diagnosis. The mean time between the diagnosis of CA-CDI and hospital admission was 1.14 days (Median: 0 days; Std. Dev: 4.87).

Of 304 CA-CDI cases, 21 (6.9%) received at least one additional prescription for metronidazole or oral vancomycin after their initial therapy; 12 cases received 1 additional prescription for metronidazole or oral vancomycin, 3 received 2 prescriptions, 4 received 3 prescriptions, and, 2 received 4 prescriptions. Of these cases, 76% were retreated within 30 days of their CDI diagnoses and 90% were retreated within 60 days.

## Discussion

Our results demonstrate that CA-CDI is occurring among populations not traditionally considered 'high-risk' (i.e., younger people, people without underlying illness, people not exposed to hospitals or antimicrobials). In fact, among our relatively young study population, the incidence rates of CA-CDI and HA-CDI were similar; 44% of all cases were community-associated.

Similar to other studies of CA-CDI, our study found that a substantial proportion of persons with CA-CDI did not have traditional risk factors for this infection: 27% did not receive any antimicrobials in the 180 days before their diagnoses and 17% did not have any of the traditional risk factors for CDI (i.e., no antimicrobial or gastric acid suppressant exposure, no underlying illness, and no history of hospitalization) [13,14]. The risk factors we identified for CA-CDI were similar to risk factors for HA-CDI. For example, prior antimicrobial use was the most common risk factor, and the 'highest-risk' antimicrobials were similar to those commonly-associated with HA-CDI (i.e., clindamycin, fluoroquinolones). Also, we found that most antimicrobials were associated with some risk for CDI and each additional antimicrobial agent increased the risk for CA-CDI further. These results support calls from organizations including the Centers for Disease Control and Prevention and the Infectious Diseases Society of America for physicians to eliminate inappropriate antimicrobial use for both inpatients and outpatients.

Our study also found that persons exposed to antimicrobial agents are at risk for CA-CDI longer than suggested by prior studies [23,24]. As expected, the highest risk for CA-CDI in our study population was within the first 30 days after the last antimicrobial exposure. But the risk remained relatively high until 60 days after exposure and did not return to baseline until 150-days after exposure.

Prior studies found that gastric acid suppressant medications increase the risk for CDI, although risk estimates for CA-CDI have varied [25-28]. In our population, use of gastric acid suppressants increased the risk for CA-CDI, even after controlling for use of antimicrobials and for gastrointestinal disease. However, most (84%) patients with CA-CDI who took gastric acid suppressants also took antimicrobials. Thus, although we did not find a statistical interaction between the effects of these medication classes, a portion of the risk attributed to gastric acid suppression may be related to concurrent antimicrobial use.

CA-CDI had economic implications in this population. Although persons in our study population did not require surgical interventions for CA-CDI, approximately one out of four cases was hospitalized, and hospitalized patients stayed an average of 4 days. Thus, our population of healthy persons with CA-CDI had about 308 hospital treatment days for these infections, which added substantially to the cost of care. Although the estimated relapse rate was relatively low, 21 (6.9%) people had relapses and received a total of 38 additional antimicrobial prescriptions for either oral vancomycin or metronidazole. Moreover, about half of the persons with CA-CDI had claims submitted for "non-specific diarrheal disease" in the month before their claims for CDI. Thus, about 150 persons experienced avoidable treatment delays and additional out-patient visits that increased the morbidity and costs associated with CDI.

Our study has several limitations. First, although the ICD-9 code for CDI has reasonable sensitivity and specificity for detecting CDI cases in inpatient settings [29,30], it has not been validated as thoroughly in community settings. We could not validate the coding in our data set because we did not have access to the patients' medical records. Future studies are needed to validate ICD-9 code 008.45 in community settings. Moreover, we could not determine either the date symptoms first occurred or the date on which *C. difficile *testing occurred. Rather, as noted previously, we had to define the date of CDI diagnosis based on the date the ICD-9 code 008.45 first appeared on insurance claims. Second, this identification strategy is contingent upon patients being tested for CDI and diagnosed in a clinical setting, thus we may have underestimated the true burden of CA-CDI. Third, we most likely underestimated the use of gastric acid suppressants because patients may purchase these medications over-the-counter. However, we presume that misclassification would be nondifferential and could attenuate the association between these agents and CA-CDI. In addition, prescription drug claims were included in our data set only if they were submitted and paid. Thus, we may have underestimated antimicrobial use if patients did not submit claims for their antimicrobial prescriptions. Fourth, inflammatory bowel disease was a significant risk factor for CA-CDI, but this association was based on only 12 cases and 4 controls. Also, patients with IBD often have diarrhea. Thus, higher rates of CA-CDI among patients with IBD than among other patients could be due to surveillance bias. Finally, the persons in our population were fully-insured through either individual or employer-based plans. In addition, the database excluded Medicare and Medicare Supplement Insurance coverage, so the number of persons 65 years or older was limited to those who also purchased commercial insurance. Thus, our results may not be generalizable to older populations. For example, the incidence of HA-CDI and of complications (i.e., none of the cases underwent colectomies) in our study may be lower than in other studies because our population was relatively young. This may also account for the low CDI relapse rate among our population.

We studied a young, healthy insured population and found the incidence of CA-CDI to be equal to that of HA-CDI, suggesting that hospital-based CDI surveillance may substantially underestimate the incidence of this infection in community settings. In general, the risk factors for CDI in our study population were similar to those identified for hospitalized populations, yet over 25% of the patients had not been exposed to antimicrobial agents. On the other hand, the population-attributable-risk percent for antimicrobial use was nearly 58% [31], indicating that over half of the cases were related to antimicrobial use, and the risk associated with antimicrobial agents persisted over several months. Moreover, it seems that many cases of CA-CDI were not diagnosed upon symptom onset, indicating that physicians may not consider this diagnosis initially. A substantial proportion of the patients were hospitalized for treatment, indicating that these infections were serious, and they increased the cost of care.

## Conclusions

Our study documented that the epidemiology of CDI is changing significantly and the population at risk for this infection is much larger than previously thought. Clinicians should be aware of these changes and obtain appropriate diagnostic testing on outpatients with diarrhea and antimicrobial exposure, including remote exposure (i.e., up to 150 days prior to disease onset). To curb spread of *C. difficile *in the community setting, we must decrease antimicrobial use among outpatients and we must conduct further research to determine the source of *C. difficile *in this setting.

## Competing interests

The authors declare that they have no competing interests.

## Authors' contributions

JLK conceived of the study, performed the statistical analysis, and drafted the manuscript. EAC, JFP, LAH, and PMP participated in the study design and contributed to the manuscript. All authors read and approved the final manuscript.

## Pre-publication history

The pre-publication history for this paper can be accessed here:

http://www.biomedcentral.com/1471-2334/11/194/prepub
